# Global research status and development trends of chronic obstructive pulmonary disease and gut microbiota: a comprehensive analysis based on bibliometrics and knowledge visualization

**DOI:** 10.3389/fmicb.2026.1836064

**Published:** 2026-06-03

**Authors:** Xinru Wu, Tianyi Zhang, Tiancheng Yu, Shuai Hu

**Affiliations:** 1 Kunshan Integrated TCM and Western Medicine Hospital, Suzhou, China; 2 Physical Education and Sports School of Soochow University, Suzhou, China; 3 Sports and Health Collaborative Innovation Center for Fitness Promotion, Jiangsu Normal University, Xuzhou, China; 4 Institute of Physical Education, Jiangsu Normal University, Xuzhou, China

**Keywords:** bibliometric analysis, gut metabolites, gut microbiota, gut-lung axis, probiotics, respiratory diseases, traditional Chinese medicine

## Abstract

**Background:**

Chronic obstructive pulmonary disease (COPD) is a common chronic disease characterized by airflow obstruction due to chronic bronchitis and/or emphysema, which can further progress to cor pulmonale and respiratory failure. In recent years, the association between gut microbiota and COPD has attracted extensive attention from researchers. This study aimed to explore the current research hotspots, knowledge gaps, and future research trends in the field of gut microbiota and COPD.

**Methods:**

A comprehensive search of literature related to gut microbiota and COPD published between 2009 and 2025 was conducted using the Web of Science and Scopus databases. Bibliometric analyses were performed using VOSviewer, CiteSpace, and R software.

**Results:**

The number of publications in this field showed a significant growth trend from 2009 to 2025, with the highest number of publications recorded in 2024. China and the United States were the leading contributing countries, and institutions such as the University of Technology Sydney made important contributions. *The International Journal of Chronic Obstructive Pulmonary Disease* served as the core publication platform in this field, and Hansbro, Philip M. was a key contributor. Research in this field involved keywords including gut-lung axis, inflammation, probiotics, bacteria, and short chain fatty acid, which revealed the core themes and trends of studies on gut microbiota and COPD.

**Conclusion:**

To our knowledge, this study presents the first quantitative bibliometric analysis of the field of gut microbiota and COPD. The core research hotspots identified include the characteristics of gut microbiota alterations in COPD patients, as well as the reciprocal interactions and underlying mechanisms between COPD and gut microbiota; microbiota intervention strategies have also emerged as an emerging research direction. Investigating immune regulation mediated by gut microbial metabolites has become an important trend in this field. This study provides a comprehensive analysis of the current research status and key hotspots in the field of gut microbiota and COPD, offering important references and insights for subsequent studies in related fields.

## 1 Introduction

Chronic obstructive pulmonary disease (COPD) is a chronic progressive respiratory disease characterized by persistent respiratory symptoms and irreversible airflow limitation. Its core pathological mechanisms are related to inflammatory response, oxidative stress, protease/antiprotease imbalance, immune imbalance, etc. (Van Der Koog et al., 2026). This disease not only leads to progressive decline in lung function and recurrent acute exacerbations in patients, but is also recognized as a systemic disease, often accompanied by a variety of extrapulmonary complications such as cardiovascular diseases and metabolic syndrome, which significantly increase the disability rate and all-cause mortality of patients and severely reduce the quality of life (Paudel et al., 2026; Qi et al., 2026). In recent years, the global disease burden of COPD has remained high, with more than 300 million people affected worldwide and over 3 million deaths annually (de Oca et al., 2025). Cigarette smoke exposure, air pollution, population aging and so on are the core driving factors for the increasing prevalence rate (Fan et al., 2026; Thawanaphong and Nair, 2025). At present, clinical interventions for COPD include lifestyle intervention, pharmacotherapy such as bronchodilators, and pulmonary rehabilitation. Among them, bronchodilators are the cornerstone of symptom management, which can alleviate airflow limitation and reduce acute exacerbations (Barnes et al., 2015; Labaki and Rosenberg, 2020). However, the existing regimens can only relieve symptoms and delay progression, cannot reverse irreversible lung function damage, and are difficult to control systemic inflammation and extrapulmonary complications, with the risk of adverse reactions during long-term medication (Xie et al., 2025). Therefore, exploring new pathogenic mechanisms of COPD and discovering novel intervention targets and therapeutic strategies have become the focus of current research in the respiratory field.

Gut microbiota is the general term for microbial communities colonizing the human gastrointestinal tract. As the largest reservoir of microorganisms and metabolites in the human body, it plays a central role in host immune development, metabolic regulation and mucosal barrier maintenance (Luo et al., 2024). The gut–lung axis, as a bidirectional signal communication system between the gastrointestinal tract and respiratory tract, is the core pathway through which gut microbiota regulates distant pulmonary immunity and pathological processes, mainly achieving cross-organ regulation through microbial metabolites, transmucosal migration of immune cells and neural signal transmission (Song et al., 2024). In recent years, the regulatory role of gut microbiota and the gut–lung axis in chronic respiratory diseases has attracted extensive attention (Xie et al., 2024). Studies have confirmed that gut microbiota and its metabolites (such as short-chain fatty acids) can regulate pulmonary immune homeostasis, inhibit systemic inflammatory cascade reactions, and maintain epithelial barrier integrity through the gut–lung axis, and these pathways are closely related to airway inflammation, lung tissue injury, disease progression and acute exacerbations in COPD (Eladham et al., 2024; Yang et al., 2025). Animal experiments have shown that microbiota-modulating interventions such as probiotics and prebiotics can improve lung function, alleviate airway inflammation and alveolar destruction, and inhibit excessive activation of the TLR4/NF-κB pro-inflammatory pathway in mouse models of COPD (Cao et al., 2023; Jia et al., 2024). Clinical studies have found that patients with COPD generally present with gut dysbiosis, characterized by decreased microbial diversity, reduced abundance of beneficial bacteria such as Bifidobacterium, and enrichment of pathogenic bacteria such as Proteobacteria (Krumina et al., 2022), and the severity of dysbiosis is significantly correlated with patients’ lung function, inflammatory level and risk of acute exacerbations (Sun et al., 2024; Wang et al., 2023). In addition, probiotic intervention can reduce systemic inflammation, decrease acute exacerbations and improve quality of life in COPD patients (Van Iersel et al., 2025; Verma et al., 2025). Gut dysbiosis can participate in the occurrence of extrapulmonary complications of COPD through systemic inflammation, and targeting the gut–lung axis provides a new idea for the prevention and treatment of its multi-system damage (Millares and Monso, 2022).

Available evidence indicates that targeting gut microbiota and the gut–lung axis has broad application prospects and translational value in the prevention and treatment of COPD, but many unsolved problems remain in the application of this strategy in long-term clinical management of COPD (Cao et al., 2024). For example, the specific molecular mechanisms and key targets of gut microbiota in regulating COPD have not been fully elucidated, probiotic effects show significant strain specificity, and there is no unified optimal intervention regimen (Ni et al., 2026; Qu et al., 2022). At present, there is a lack of systematic analysis of research hotspots and frontier trends in the field of gut microbiota and COPD, which is not conducive for researchers to quickly and accurately grasp the development context and future research directions of this field.

As a mature scientific quantitative analysis tool, bibliometrics can systematically reveal the development history, research hotspots, frontier dynamics and knowledge structure of a disciplinary field through statistical analysis of scientific and technological literature in a specific field (Xi et al., 2026). Applying bibliometric methods to research related to gut microbiota in COPD can not only comprehensively sort out the development status and research context of this field, but also accurately identify current research gaps and future development directions, providing important references and theoretical guidance for subsequent basic mechanistic research and clinical translational applications. Therefore, this study adopts bibliometric methods to conduct a systematic and comprehensive quantitative analysis of relevant research literature in the field of gut microbiota and COPD, aiming to provide valuable references and research ideas for researchers and clinicians in this field, and promote the standardized application and in-depth research of gut microbiota-targeted COPD management.

## 2 Materials and methods

### 2.1 Literature sources and search strategy

This study conducted literature searches in the Web of Science Core Collection (WoSCC) and Scopus databases on March 16, 2026. WoSCC search strategy: TS = (“COPD” OR “chronic obstructive pulmonary disease*”) AND TS = (“gut microb*” OR “intestinal microb*” OR “gut microflora” OR “intestinal microflora” OR “gut microorganism” OR “intestinal microorganism” OR “probiotics” OR “prebiotics” OR “synbiotics”) AND PY = (2009–2025) AND DT = (Article OR Review) AND LA = (English). Scopus search strategy: TITLE-ABS-KEY(“COPD” OR “chronic obstructive pulmonary disease”) AND TITLE-ABS-KEY(“gut microbiota” OR “gut microbiome” OR “intestinal microbiota” OR “intestinal microbiome” OR “gut flora” OR “intestinal flora”) AND PUBYEAR > 2008 AND PUBYEAR < 2026 AND (LIMIT-TO (DOCTYPE, “ar”) OR LIMIT-TO (DOCTYPE, “re”)) AND (LIMIT-TO (LANGUAGE, “English”)). Records retrieved from WoSCC were saved in plain text format, with full records including references exported; records retrieved from Scopus were saved in CSV format, and full records including references were also exported.

### 2.2 Inclusion and exclusion criteria

**Figure 1:**
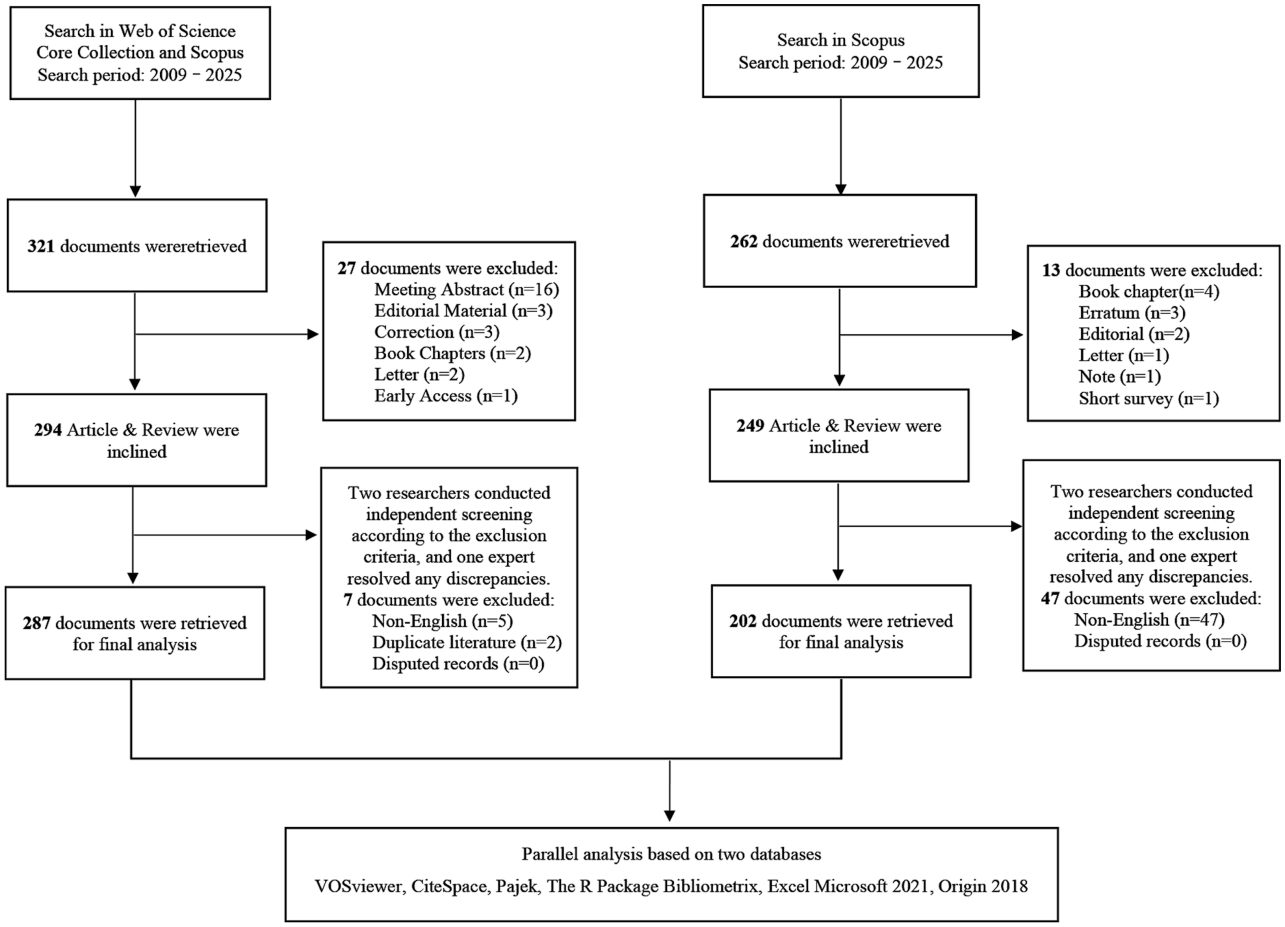
Flowchart of the publications selection.

The preset inclusion criteria of this study were all original English research articles and review literature exploring the association between gut microbiota and COPD. The exclusion criteria were as follows: duplicate documents were removed using NoteExpress software (Aegean Software Company, Beijing, China) (WoSCC, *n* = 2; Scopus, *n* = 0); literature irrelevant to the core topics of gut microbiota and COPD (including those only mentioning COPD incidentally or focusing on other pulmonary diseases as the main research subject); conference abstracts, conference proceedings, pre-published online-ahead-of-print articles, editorial commentaries, book chapters, retracted papers, and letters to the editor. To ensure the accuracy of research data and reproducibility of results, all data extraction was independently performed by two researchers. Any discrepancies between the two researchers were resolved by submitting the controversial records to a third senior researcher for independent evaluation and final decision. After systematic retrieval and stepwise screening, a total of 287 eligible articles were finally included from the WoSCC database and 202 eligible articles from the Scopus database. The complete process of literature retrieval and screening is shown in Figure 1.

### 2.3 Data analysis

This study adopted a well-established methodological framework. Origin 2018 was used to analyze the annual publication trends, while R software (version 4.5.1) combined with the Bibliometrix package (version 4.01), VOSviewer (version 1.6.20), and CiteSpace (version 6.1.4) was employed for analyses. Specifically, the Bibliometrix package was applied for visual analysis and mapping of scientific knowledge, and VOSviewer was used to visualize co-authorship networks at the country and institution levels, co-citation relationships of source documents, and keyword co-occurrence patterns. Given the inherent differences in data formats between the WoSCC and Scopus databases, merging the two datasets may easily lead to information loss. Therefore, independent analyses were separately conducted on data from the two databases to ensure the stability and reliability of the research findings.

In the co-authorship network analyses of the WoSCC and Scopus datasets, VOSviewer software was used to generate maps of national collaboration networks, co-citation analysis maps of source journals, and visual maps of keyword co-occurrence. The relevant parameter settings were as follows: the minimum publication threshold for countries was set at ≥2 and ≥1 publications; the minimum publication threshold for institutions was set at ≥3 and ≥1 publications; the minimum citation threshold for authors was set at ≥3 and ≥2 citations; in the co-citation analysis of source journals, the minimum citation threshold for journals was set at ≥23 and ≥22 citations; in keyword co-occurrence analysis, the minimum occurrence threshold for keywords was set at ≥5 and ≥6 occurrences. CiteSpace software was used to identify the top 25 most highly cited references in this field, with the following parameter settings: time slicing ranged from 2009 to 2025, the duration of each time slice was 1 year, node type was selected as cited references, the selection criterion was set as top N = 50, and no pruning was performed. Journal impact factor (IF) data used in this study were all derived from the 2024 Journal Citation Reports (JCR). Basic keywords such as “gut microbiota,” “chronic obstructive pulmonary disease,” and their synonyms were excluded from the analyses.

## 3 Results

### 3.1 General overview of gut microbiota and COPD

**Figure 2:**
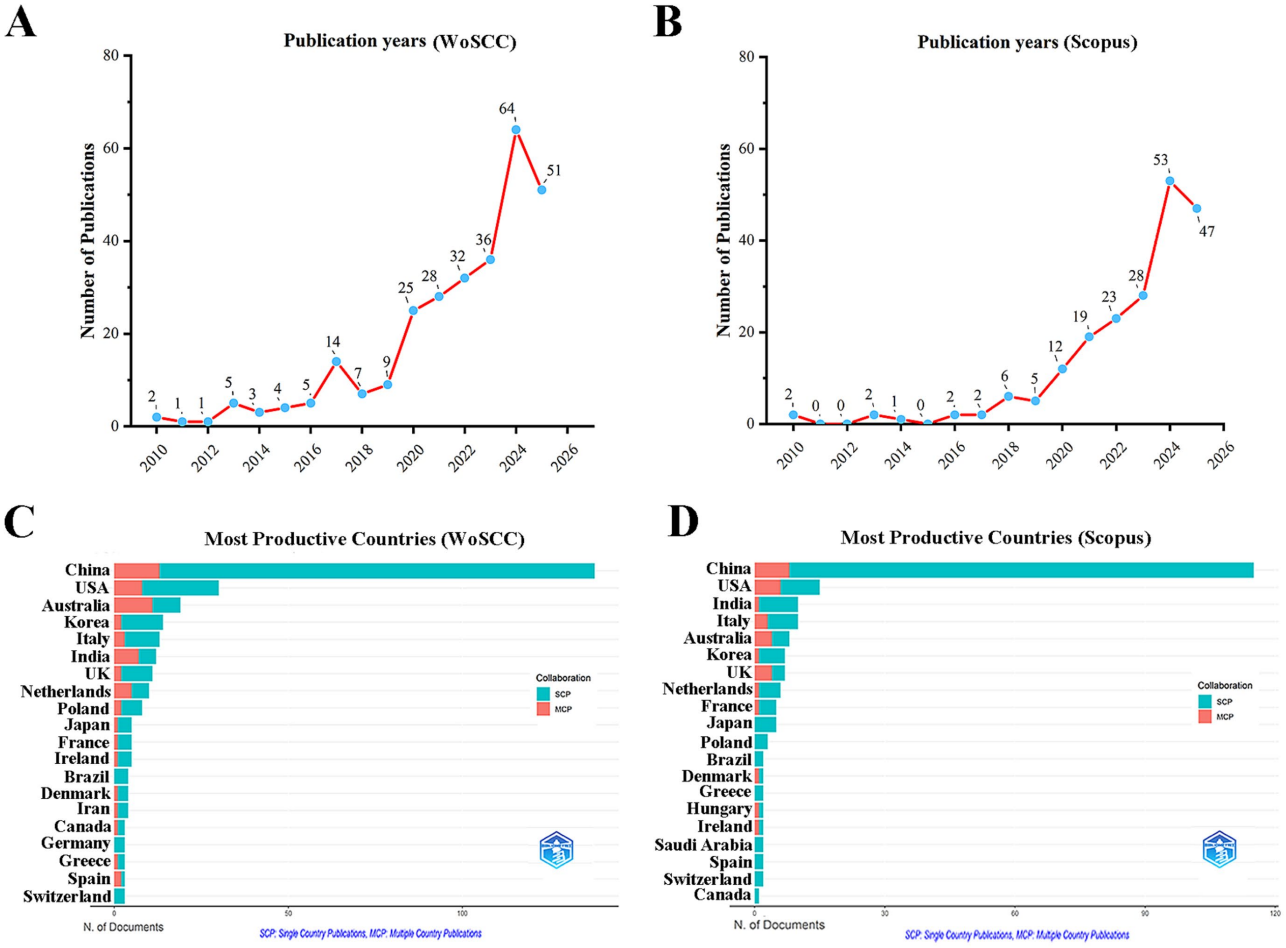
Annual publication trends on the relationship between gut microbiota and COPD from 2009 to 2025. **(A)** Annual publication trends in WoSCC. **(B)** Annual publication trends in Scopus. **(C)** Distribution of corresponding authors’ countries in WoSCC. **(D)** Distribution of corresponding authors’ countries in Scopus.

A total of 287 independent articles were retrieved from the WoSCC database. The annual publication output in this field showed an overall upward trend from 2009 to 2025, with a rapid increase especially after 2019, reaching a peak of 64 publications in 2024. A total of 202 deduplicated independent records were obtained from the Scopus database, with a publication trend consistent with that of the WoSCC database, indicating a sustained growing research interest in the association between the two (Figures 2A,B).

**Figure 3:**
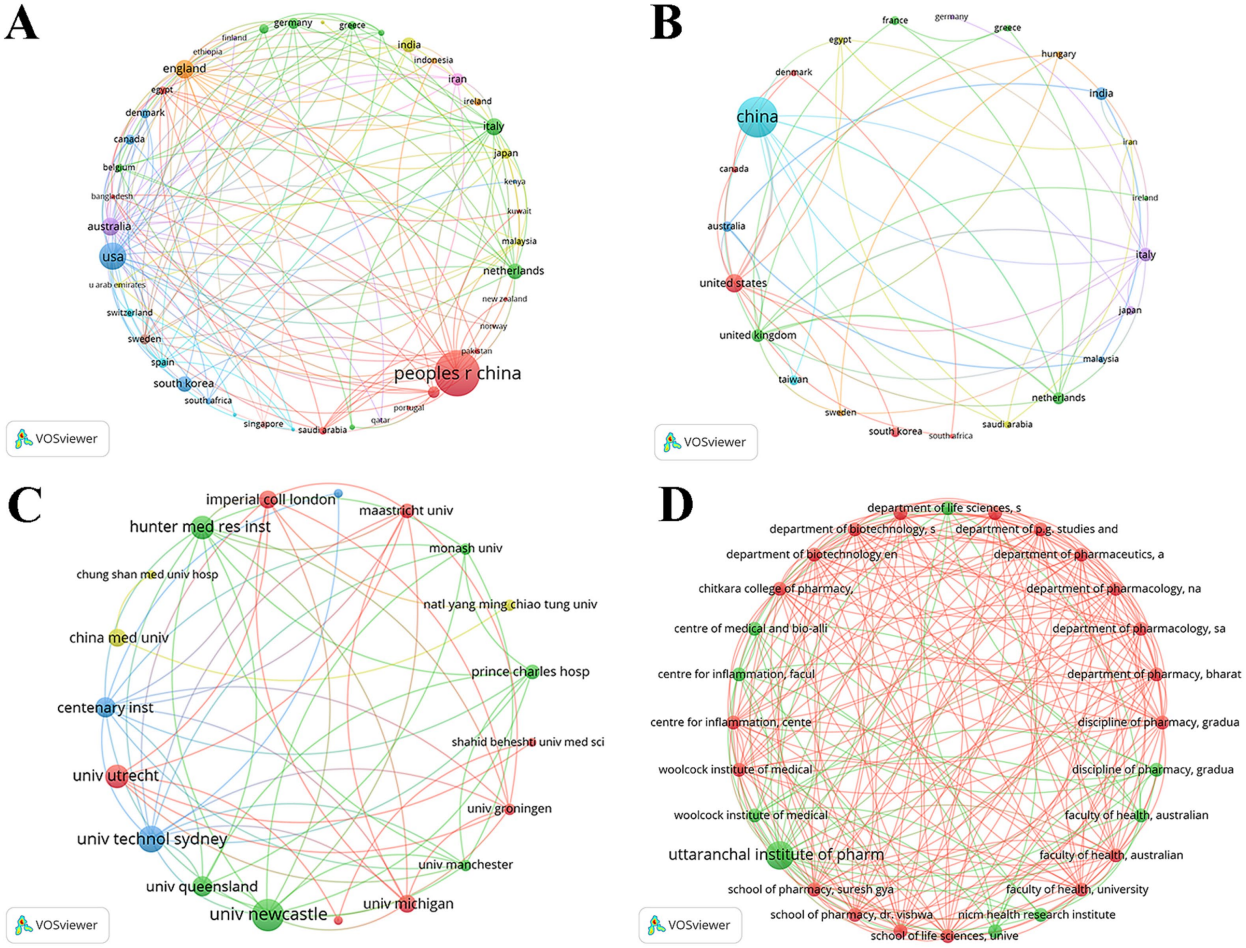
Global mapping of countries regions and institutions in gut microbiota and COPD research from 2009 to 2025. **(A)** Geospatial collaboration network of countries based on WoSCC. **(B)** Geospatial collaboration network of countries based on Scopus. **(C)** Inter-institutional collaboration network derived from WoSCC. **(D)** Inter-institutional collaboration network derived from Scopus.

National contribution analysis revealed that in the WoSCC database, China ranked first in the number of publications (*n* = 134), followed by the United States (*n* = 24) and Australia (*n* = 17). Among them, 9% of publications from China and 25% from the US were international collaborative outputs (Figure 2C; Table 1). In the Scopus database, China still ranked first (*n* = 115), followed by the United States (*n* = 15) and India (*n* = 10). Of these, 7% of Chinese publications and 40% of US publications were results of international cooperation (Figure 2D; Table 1). In addition to leading publication volume, China serves as a central hub in the collaborative network of this field and plays a key leading role in its development (Figures 3A,B). Overall, China holds a leading position in research related to gut microbiota and COPD, which may be related to the large population affected and severity of COPD in China (Figure 3B, Table 2).

**Table 1:** Most relevant countries by corresponding authors.

Country	Articles	SCP	MCP	Freq (%)	MCP_Ratio (%)
WoSCC					
China	134	122	12	46.4	9
United States	24	18	6	8.3	25
Australia	17	7	10	5.9	58.8
Korea	14	12	2	4.8	14.3
Italy	12	10	2	4.2	16.7
India	10	8	2	3.5	20
United Kingdom	9	3	6	3.1	66.7
Netherlands	8	4	4	2.8	50
Poland	7	5	2	2.4	28.6
Japan	5	4	1	1.7	20
Scopus					
China	115	107	8	52.5	7
United States	15	9	6	6.8	40
India	10	9	1	4.6	10
Italy	10	7	3	4.6	30
Australia	8	4	4	3.7	50
Korea	7	6	1	3.2	14.3
United Kingdom	7	3	4	3.2	57.1
Netherlands	6	5	1	2.7	16.7
France	5	4	1	2.3	20
Japan	5	5	0	2.3	0

MCP, Multiple country publication; SCP, Single country publication.

**Table 2:** Top 10 most relevant affiliations of gut microbiota and COPD.

WoSCC		Scopus	
Affiliation	Articles (n)	Affiliation	Articles (n)
University of Technology Sydney	26	University of Technology Sydney	10
University of Newcastle	23	University of Pittsburgh	9
Guangzhou Medical University	22	Henan University of Chinese Medicine	8
Chengdu University of Traditional Chinese Medicine	18	Southern Medical University	8
University of Queensland	18	Fudan University	7
University of Sydney	18	Jilin University	7
University of Ulsan	17	Nanjing University of Chinese Medicine	7
University of California System	15	Chengdu University of Traditional Chinese Medicine	6
Pennsylvania Commonwealth System of Higher Education (Pcshe)	14	Hospital of Chengdu University of Traditional Chinese Medicine	6
Taipei Medical University	14	Imperial College London	6

Institutional analysis further showed that in the WoSCC database, the University of Technology Sydney in Australia had the highest number of publications (*n* = 26), followed by the University of Newcastle in the UK (*n* = 23). In the Scopus database, the University of Technology Sydney ranked first (10 publications), followed by the University of Pittsburgh in the US (9 publications) (Table 2). In terms of collaborative networks, the University of Technology Sydney and the University of Newcastle act as core collaborative hubs (Figures 3C,D). In summary, the University of Technology Sydney not only produces a high volume of publications but also maintains an extensive international collaborative network in this field.

### 3.2 Journals and co-cited journals

**Figure 4:**
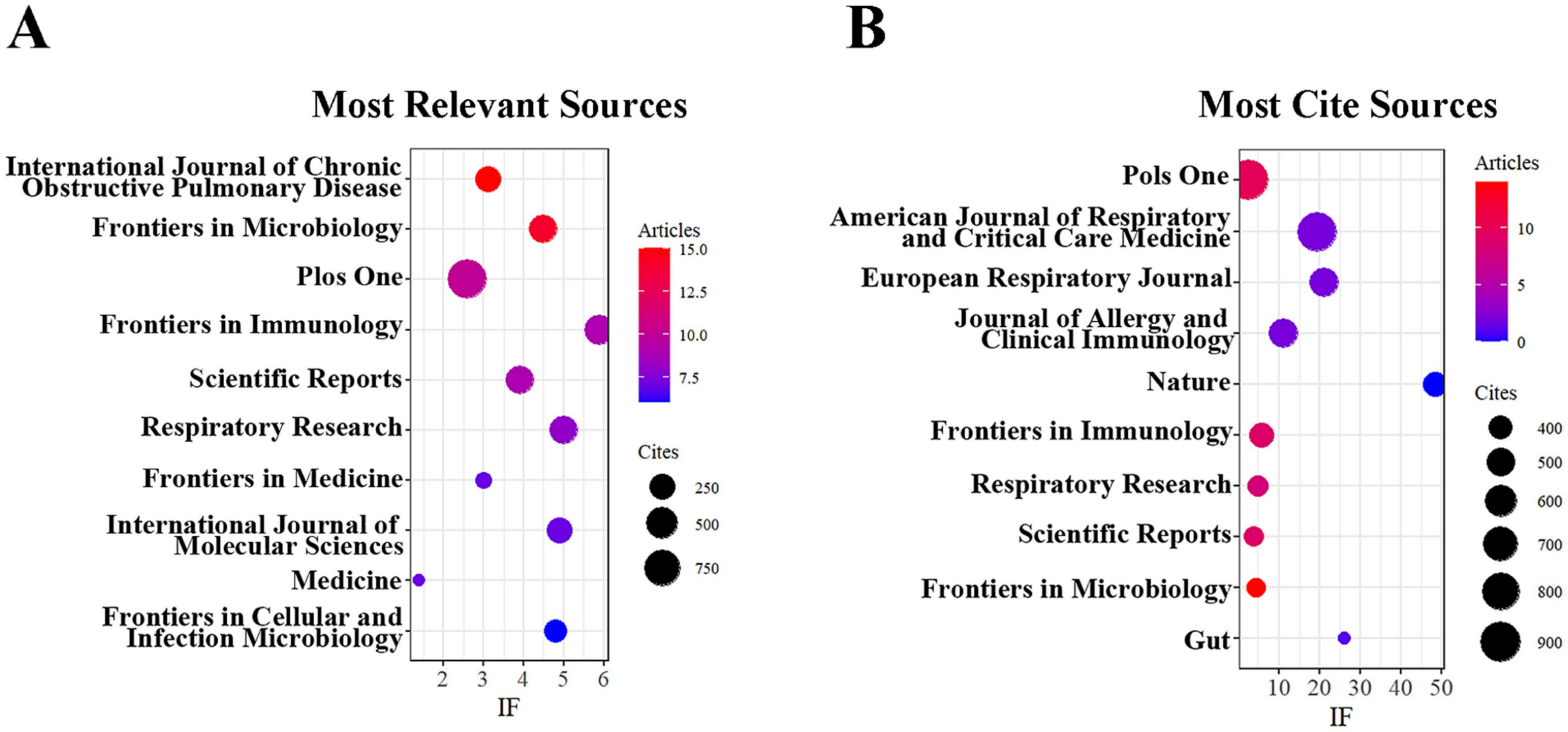
Journal with the largest number of articles published and the journal with the largest number of citations (WoSCC). **(A)** Journal with the largest number of articles published. **(B)** Journals with the largest number of citations.

To evaluate journal influence, this study used Bibliometrix for outcome analysis, ggplot2 for visualization, and VOSviewer to construct co-citation maps. The results showed that the 287 articles from the WoSCC database were distributed across 185 academic journals (Supplementary material 1). As shown in Table 3 and Figure 4A, the journal with the highest publication count was the *International Journal of Chronic Obstructive Pulmonary Disease* (*n* = 15, IF = 3.1), followed by *Frontiers in Microbiology* (*n* = 14, IF = 4.5), *PLOS ONE* (*n* = 10, IF = 2.6), *Respiratory Research* (*n* = 8, IF = 5), and *Frontiers in Immunology* (*n* = 7, IF = 5.9). Table 4 and Figure 4B present the most frequently cited journals, including *PLOS ONE* (*n* = 912, IF = 2.6), *American Journal of Respiratory and Critical Care Medicine* (*n* = 864, IF = 19.4), *Journal of Allergy and Clinical Immunology* (*n* = 526, IF = 14.2), *European Respiratory Journal* (*n* = 523, IF = 21.2), and *Nature* (*n* = 421, IF = 48.5).

**Table 3:** Top 10 journals with the most published articles.

WoSCC				Scopus		
Journal	Articles	Cites	IF (2024)	Journal	Articles	IF (2024)
International Journal of Chronic Obstructive Pulmonary Disease	15	697	3.1	International Journal of Chronic Obstructive Pulmonary Disease	13	3.1
Frontiers in Microbiology	14	387	4.5	Frontiers in Microbiology	11	4.5
Plos One	10	276	2.6	Frontiers in Immunology	7	5.9
Respiratory Research	8	245	5.0	Respiratory Research	7	5
Frontiers in Immunology	7	169	5.9	Scientific Reports	7	3.9
International Journal of Molecular Sciences	7	699	4.9	Plos One	6	2.6
Scientific Reports	7	957	3.9	Frontiers in Cellular And Infection Microbiology	5	4.8
Frontiers in Cellular and Infection Microbiology	6	352	4.8	Frontiers in Medicine	5	3
Frontiers in Medicine	6	313	3	International Journal of Molecular Sciences	4	4.9
Medicine	6	388	1.4	Journal of Ethnopharmacology	4	5.4

**Table 4:** Top 10 journals with the most cited journals (WoSCC).

Rank	Journal	Cites	Articles	IF (2024)
1	PLoS One	912	10	2.6
2	American Journal of Respiratory and Critical Care Medicine	864	2	19.4
3	Journal of Allergy and Clinical Immunology	526	2	14.2
4	European Respiratory Journal	523	2	21.2
5	Nature	421	0	48.5
6	Frontiers in Immunology	396	7	5.9
7	Respiratory Research	344	8	5.0
8	Scientific Reports	328	7	2.6
9	Frontiers In Microbiology	321	14	4.5
10	Gut	309	2	26.2

The 202 articles from the Scopus database were distributed across 137 academic journals (Supplementary material 2). As shown in Table 3, the leading journal was the *International Journal of Chronic Obstructive Pulmonary Disease* (*n* = 13, IF = 3.1), followed by *Frontiers in Microbiology* (*n* = 11, IF = 4.5), *Frontiers in Immunology* (*n* = 7, IF = 5.9), *Respiratory Research* (*n* = 7, IF = 5), and *Scientific Reports* (*n* = 7, IF = 3.9).

**Figure 5:**
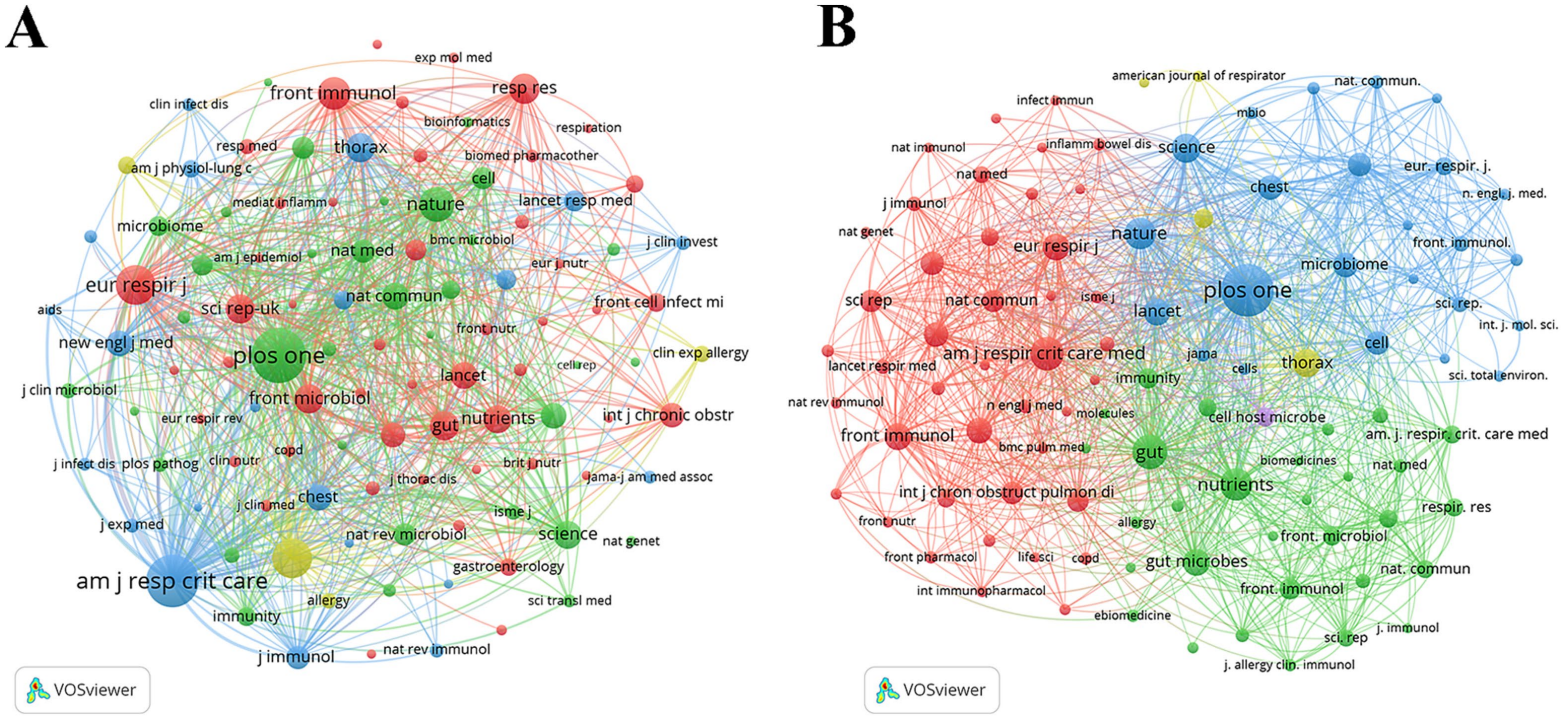
Co-cited journals related to gut microbiota and COPD. **(A)** Journal co-citation network based on WoSCC. **(B)** Journal co-citation network based on Scopus.

Notably, the journal co-citation networks of both the WoSCC and Scopus databases (Figures 5A,B) demonstrated that *PLOS ONE* was a central hub in the co-citation structure. These findings indicate that the *International Journal of Chronic Obstructive Pulmonary Disease* and *PLOS ONE* exert significant academic influence in the field of gut microbiota and COPD research.

### 3.3 Authors and co-authors

Table 5 lists the top 10 most productive authors in the field. In the WoSCC database, the top three were Hansbro, Philip M. (*n* = 11), Budden, Kurtis F. (*n* = 6), and Lee, Sei Won (*n* = 6). Table 6 presents the top 10 authors by total citation frequency, with the top three being Hansbro, Philip M. (*n* = 181), Budden, Kurtis F. (*n* = 141), and Shukla, Shakti D (*n* = 134).

**Table 5:** Top 10 documents authors related to gut microbiota and COPD.

WoSCC			Scopus		
Authors	Documents	Citations	Authors	Documents	Citations
Hansbro, Philip M.	11	181	Hansbro, Philip M.	5	102
Budden, Kurtis F.	6	141	Lee, Se Hee	4	54
Lee, Sei Won	6	33	Lee, Sei Won	4	54
Morris, Alison	6	4	Li, Bing	4	80
Folkerts, Gert	5	67	Morris, Alison	4	3
Garssen, Johan	5	67	Pu, Jinding	4	80
Gellatly, Shaan L.	5	122	Ran, Pixin	4	80
Hugenholtz, Philip	5	122	Wang, Jing	4	16
Shukla, Shakti D.	5	134	Ding, Jianbing	3	14
Zhang, Jing	5	13	Jiang, Min	3	14

**Table 6:** Top 10 citations authors related to gut microbiota and COPD (WoSCC).

Rank	Authors	Citations	Documents
1	Hansbro, Philip M.	181	10
2	Budden, Kurtis F.	141	6
3	Shukla, Shakti D.	134	5
4	Vaughan, Annalicia	129	4
5	Yang, Ian A.	124	4
6	Gellatly, Shaan L.	122	5
7	Hugenholtz, Philip	122	5
8	Wood, David L. A.	118	4
9	Bowerman, Kate L.	98	2
10	Lachner, Nancy	98	2

In the Scopus database, Hansbro, Philip M. had the highest publication count (*n* = 5), followed by Lee, Se Hee (*n* = 4) and Lee, Sei Won (*n* = 4). Among the top 10 authors by total citations, the top five were Hansbro, Philip M. (*n* = 102), Vaughan, Annalicia (*n* = 92), Yang, Ian A. (*n* = 92), Li, Bing (*n* = 80), and Pu, Jinding (*n* = 80).

**Figure 6:**
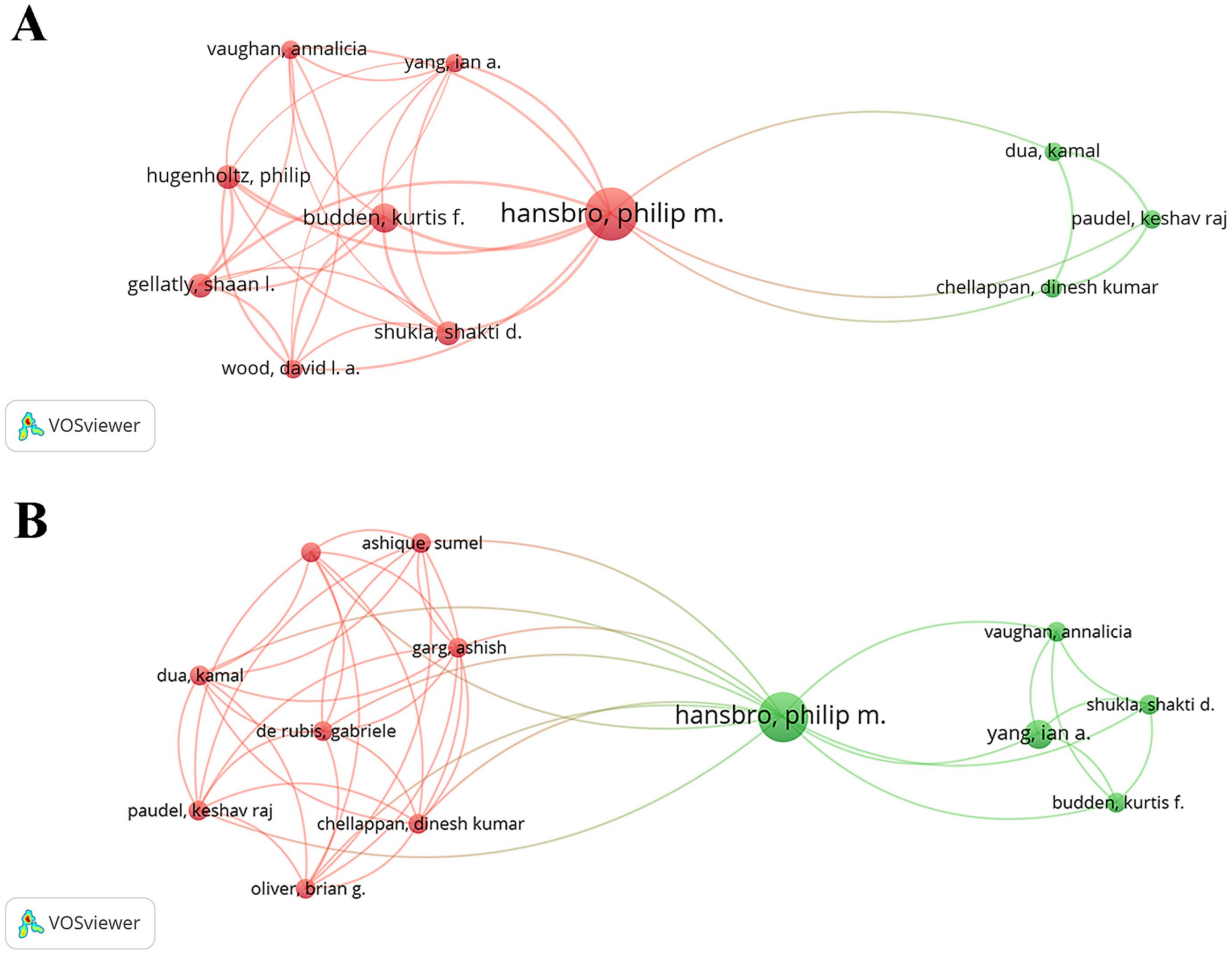
The map of co-authorship in the field of gut microbiota and COPD. **(A)** Co-authorship network based on WoSCC. **(B)** Co-authorship network based on Scopus.

Author co-authorship networks from the WoSCC and Scopus databases (Figure 6) revealed extensive and close collaboration among researchers in the field, with Hansbro, Philip M. as the central node, reflecting his profound impact on the discipline.

### 3.4 Most cited references and citation bursts

Using the bibliometrix package in R software, this study systematically identified the top 25 most highly cited articles in the field of COPD, gut microbiota, and related research. In the WoSCC database, each of the top 25 highly cited papers was cited more than 113 times (Table 7). The most frequently cited articles were *The immune response to Prevotella bacteria in chronic inflammatory disease*, *Proteobacteria: A Common Factor in Human Diseases*, and *The Continuum of Aging and Age-Related Diseases: Common Mechanisms but Different Rates*.

**Table 7:** Top 25 cited references related to gut microbiota and COPD (WoSCC).

Paper	DOI	Total Citations	TC per Year
LARSEN JM, 2017, IMMUNOLOGY	10.1111/imm.12760	1,071	107.10
RIZZATTI G, 2017, BIOMED RES INT	10.1155/2017/9351507	1,026	102.60
FRANCESCHI C, 2018, FRONT MED-LAUSANNE	10.3389/fmed.2018.00061	658	73.11
SEGAL LN, 2016, NAT MICROBIOL	10.1038/NMICROBIOL.2016.31	529	48.09
ZHANG DP, 2020, FRONT MICROBIOL	10.3389/fmicb.2020.00301	321	45.86
BOWERMAN KL, 2020, NAT COMMUN	10.1038/s41467-020-19701-0	309	44.14
BECK JM, 2012, TRANSL RES	10.1016/j.trsl.2012.02.005	280	18.67
LI RM, 2024, SIGNAL TRANSDUCT TAR	10.1038/s41392-023-01722-y	268	89.33
LAI HC, 2022, GUT	10.1136/gutjnl-2020-322599	260	52.00
HUANG CR, 2019, J TRANSL MED	10.1186/s12967-019-1971-7	238	29.75
RASTOGI S, 2022, FRONT PHARMACOL	10.3389/fphar.2022.1042189	233	46.60
SHUKLA SD, 2017, CLIN TRANSL IMMUNOL	10.1038/cti.2017.6	226	22.60
LI CX, 2020, J IMMUNOL RES	10.1155/2020/2340670	191	27.29
FANER R, 2017, EUR RESPIR J	10.1183/13993003.02086-2016	187	18.70
BENGMARK S, 2013, PHARMACOL RES	10.1016/j.phrs.2012.09.002	179	12.79
LARSEN JM, 2015, IMMUNOLOGY	10.1111/imm.12376	157	13.08
HANSSON GC, 2019, J INTERN MED	10.1111/joim.12910	151	18.88
WANG L, 2023, AM J RESP CRIT CARE	10.1164/rccm.202206-1066TR	149	37.25
CRIBBS SK, 2020, PHYSIOL REV	10.1152/physrev.00039.2018	139	19.86
LI NJ, 2021, RESP RES	10.1186/s12931-021-01872-z	135	22.50
RAHMAN MM, 2022, BIOMED PHARMACOTHER	10.1016/j.biopha.2022.113041	130	26.00
RAFTERY AL, 2020, FRONT IMMUNOL	10.3389/fimmu.2020.02144	128	18.29
MA PJ, 2022, BIOMED PHARMACOTHER	10.1016/j.biopha.2022.113810	124	24.80
MAHDAVINIA M, 2016, CLIN EXP ALLERGY	10.1111/cea.12666	115	10.45
LIN TL, 2020, FRONT PHARMACOL	10.3389/fphar.2020.00554	113	16.14

In the Scopus database, each of the top 25 highly cited papers was cited more than 72 times (Table 8). The top cited articles were *Intestinal Microbiome Shifts, Dysbiosis, Inflammation, and Non-alcoholic Fatty Liver Disease*, *The Cross-Talk Between Gut Microbiota and Lungs in Common Lung Diseases*, and *Disease-associated gut microbiome and metabolome changes in patients with chronic obstructive pulmonary disease*.

**Table 8:** Top 25 cited references related to gut microbiota and COPD (Scopus).

Paper	DOI	Total Citations	TC per Year
FRANCESCHI C, 2018, FRONT MED	10.3389/fmed.2018.00061	742	82.44
ZHANG D, 2020, FRONT MICROBIOL	10.3389/fmicb.2020.00301	366	52.29
BOWERMAN KL, 2020, NAT COMMUN	10.1038/s41467-020-19701-0	331	47.29
LI R, 2024, SIGNAL TRANSDUCT TARGET THER	10.1038/s41392-023-01722-y	274	91.33
HUANG C, 2019, J TRANSL MED	10.1186/s12967-019-1971-7	265	33.13
LAI H-C, 2022, GUT	10.1136/gutjnl-2020-322599	261	52.20
RASTOGI S, 2022, FRONT PHARMACOL	10.3389/fphar.2022.1042189	232	46.40
CHUNXI L, 2020, J IMMUNOL RES	10.1155/2020/2340670	224	32.00
BENGMARK S, 2013, PHARMACOL RES	10.1016/j.phrs.2012.09.002	214	15.29
WANG L, 2023, AM J RESPIR CRIT CARE MED	10.1164/rccm.202206-1066TR	151	37.75
LI N, 2021, RESPIR RES	10.1186/s12931-021-01872-z	145	24.17
RAFTERY AL, 2020, FRONT IMMUNOL	10.3389/fimmu.2020.02144	133	19.00
MA P-J, 2022, BIOMED PHARMACOTHER	10.1016/j.biopha.2022.113810	129	25.80
ASHIQUE S, 2022, CHEM-BIOL INTERACT	10.1016/j.cbi.2022.110231	129	25.80
SAINT-CRIQ V, 2021, AGEING RES REV	10.1016/j.arr.2020.101235	107	17.83
BARFOD KK, 2013, BMC MICROBIOL	10.1186/1471-2180-13-303	102	7.29
JANG YO, 2020, EXP MOL MED	10.1038/s12276-020-0469-y	100	14.29
VAUGHAN A, 2019, J THORAC DIS	10.21037/jtd.2019.10.40	92	11.50
ALEMAO CA, 2021, ALLERGY EUR J ALLERGY CLIN IMMUNOL	10.1111/all.14548	88	14.67
GOLLWITZER ES, 2014, PHARMACOL THER	10.1016/j.pharmthera.2013.08.002	82	6.31
DE SIRE R, 2018, MINERVA GASTROENTEROL DIETOL	10.23736/S1121-421X.18.02511-4	81	9.00
JANG YO, 2021, SCI REP	10.1038/s41598-021-86404-x	79	13.17
QU L, 2022, FRONT MICROBIOL	10.3389/fmicb.2022.868086	78	15.60
ÖZÇAM M, 2024, NAT REV MICROBIOL	10.1038/s41579-024-01048-8	74	24.67
MENDEZ R, 2019, IUBMB LIFE	10.1002/iub.1969	72	9.00

**Figure 7:**
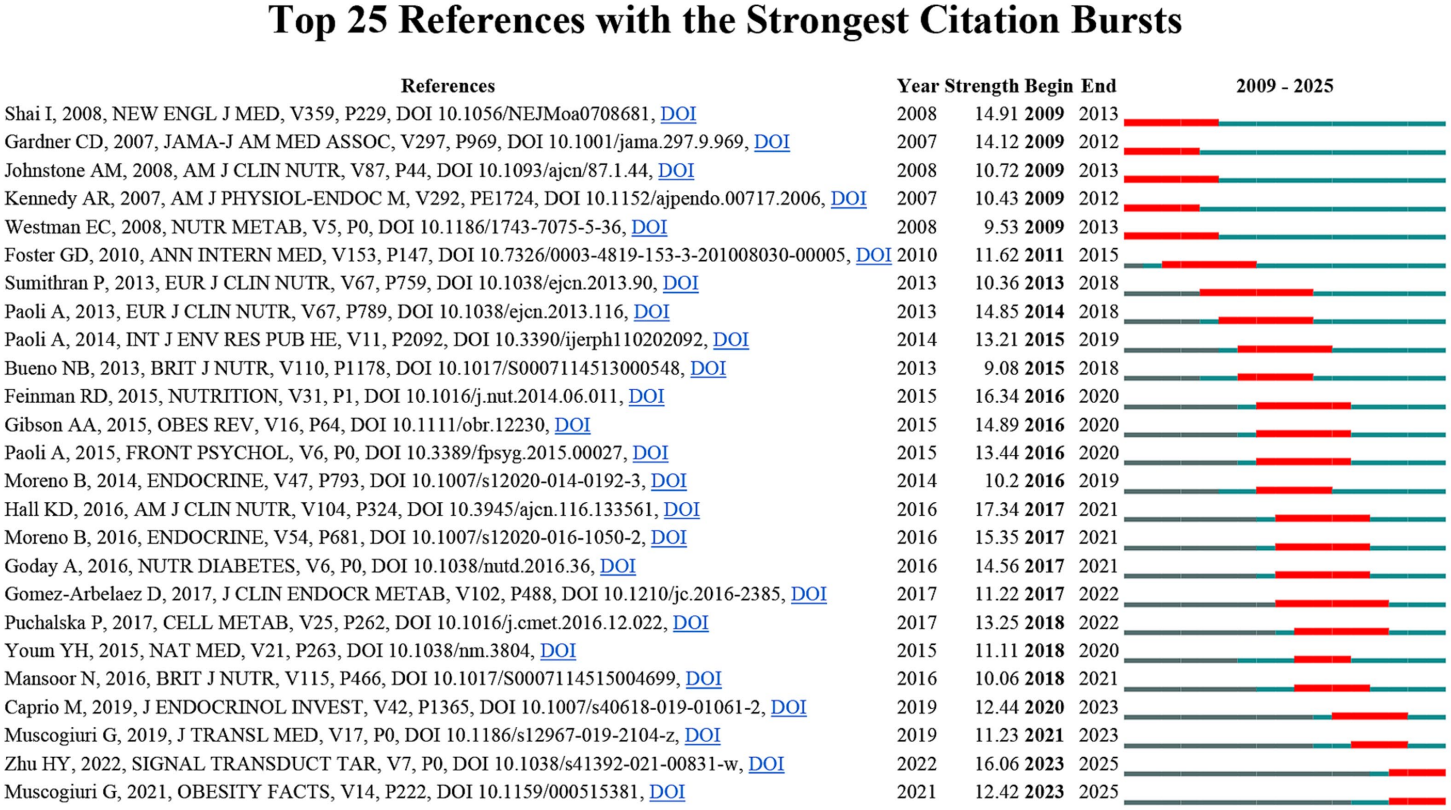
Top 25 references with the strongest citation bursts in gut microbiota and COPD (WoSCC).

CiteSpace analysis showed that 25 articles in the WoSCC database exhibited citation bursts. The three articles with the strongest burst strengths were *Emerging pathogenic links between microbiota and the gut-lung axis* (burst strength = 12.79), *The lung tissue microbiome in chronic obstructive pulmonary disease* (burst strength = 8.95), and *Microbes, metabolites, and the gut-lung axis* (burst strength = 6.86). In addition, the three most recently published articles among these high-burst-strength publications were Gut microbiota dysbiosis contributes to the development of chronic obstructive pulmonary disease, Chronic obstructive pulmonary disease, and The Bidirectional Gut-Lung Axis in Chronic Obstructive Pulmonary Disease (Figure 7).

Comprehensive analysis of highly cited literature shows that as academic attention to COPD prevention and management continues to rise, gut microbiota has become an important target for COPD. However, key issues such as the standardized clinical application of gut microbiota in COPD treatment and underlying mechanisms of action remain incompletely elucidated, warranting further in-depth investigations.

### 3.5 Keyword and thematic trend analysis

**Figure 8:**
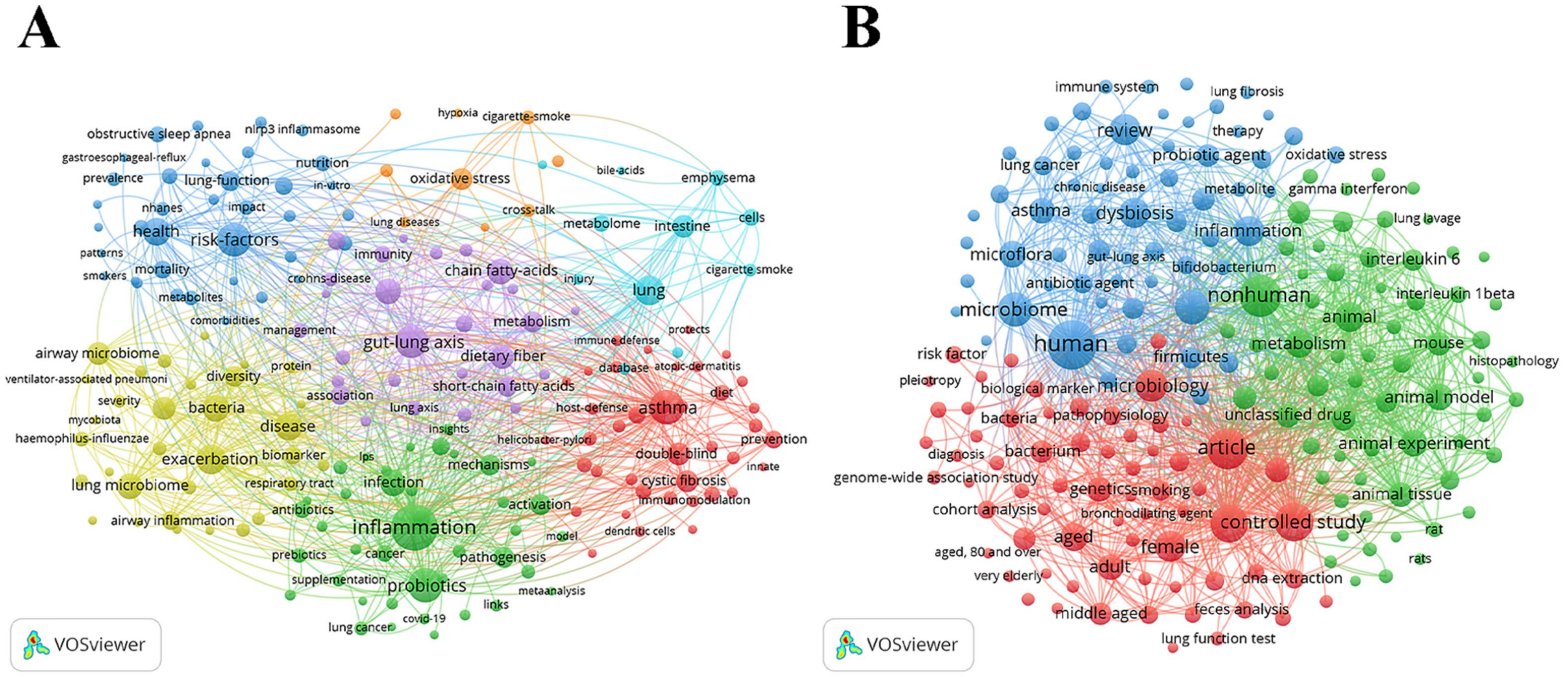
Keyword co-occurrence map of publications in gut microbiota and COPD. **(A)** Keyword co-occurrence analysis based on WoSCC. **(B)** Keyword co-occurrence analysis based on Scopus.

High-frequency keywords reflect cutting-edge dynamics in a specific knowledge domain. Using VOSviewer software, this study identified 1,653 keywords in the WoSCC database. The top 10 high-frequency keywords listed in Table 9 all appeared ≥27 times, with “inflammation” being the most frequent (*n* = 77), followed by “asthma” (*n* = 48) and “probiotics” (*n* = 41). Subsequently, based on a threshold of keyword occurrence ≥3, 201 keywords were selected to construct a keyword clustering map (Figure 8), revealing seven distinct color-coded clusters. Red Cluster 1 focused on the effects of gut microbiota on respiratory health via immune regulatory axes, comprising 40 keywords including immune-response, intestinal permeability, *Porphyromonas gingivalis*, *Lactobacillus casei*, extracellular vesicles, allergic, placebo-controlled trial, and treatment. Green Cluster 2 centered on mechanisms and therapeutic effects of microbiota-based interventions for COPD-related inflammation and respiratory diseases, covering 40 keywords such as inflammation, lung inflammation, gut-lung axis, butyrate, probiotics, supplementation, chronic respiratory diseases, influenza, and nanoparticles. Blue Cluster 3 explored environmental and dietary influences on gut microbiota and lung function in COPD, including 36 keywords such as particulate matter, exposure, dietary patterns, dietary index for gut microbiota, anti-inflammation, lung-function, and health. Yellow Cluster 4 focused on the role of respiratory microbiome dysbiosis in COPD exacerbations, with 32 keywords including COPD exacerbation, severity, sputum microbiome, dysbiosis, diversity, *Pseudomonas aeruginosa*, airway inflammation, and dynamics. Purple Cluster 5 investigated mechanisms of short-chain fatty acids in mediating the gut-lung axis in COPD interventions, containing 28 keywords such as interplay, short-chain fatty acids, fecal microbiota transplantation, acute lung injury, management, and epidemiology. Light Blue Cluster 6 addressed the effects of bile acid metabolism on emphysema, including 11 keywords such as emphysema, injury, bile-acids, intestine, insulin-resistance, metabolome, and network pharmacology. Orange Cluster focused on oxidative stress mechanisms underlying cigarette smoke-induced intestinal barrier damage in COPD, with 9 keywords including cigarette-smoke, oxidative stress, epithelial barrier function, gene-expression, hypoxia, and lung diseases.

**Table 9:** Top 10 keywords related to gut microbiota and COPD.

WoSCC		Scopus	
Keyword	Count	Keyword	Count
Inflammation	77	Human	164
Asthma	48	Nonhuman	119
Probiotics	41	Article	115
Gut-lung axis	39	Controlled study	99
Risk-factors	37	Male	93
Health	30	Lung	78
Disease	29	Microbiology	71
Exacerbation	27	Female	66
Lung	27	Animals	64

A total of 3,710 keywords were identified from the Scopus database using VOSviewer. The top 10 keywords with occurrence ≥64 times are listed in Table 9, reflecting major research priorities in this field. “Human” was the most frequent (164 times), followed by “nonhuman” (119 times), “article” (115 times), “controlled study” (99 times), and “male” (93 times). Based on a keyword occurrence threshold ≥9, 195 keywords were selected to generate a keyword clustering map (Figure 8B), yielding three distinct clusters. Red Cluster 1 focused on clinical associations and interactions of gut microbiota biomarkers in COPD, including 68 keywords such as article, controlled study, microbiology, female, biological marker, clinical article, disease severity, disease association, observational study, and clinical trial. Green Cluster 2 investigated the effects and molecular mechanisms of gut microbiota-derived short-chain fatty acids in improving airway inflammation and lung injury in COPD, covering 65 keywords such as animal experiment, metabolism, lung function, short chain fatty acid, interleukin 6, tumor necrosis factor, toll like receptor 4, protein expression, and gene expression. Blue Cluster 3 explored immune-inflammatory regulatory mechanisms and intervention strategies of gut microbiota dysbiosis in COPD, including 62 keywords such as immune response, inflammation, gut-lung axis, probiotic agent, lung microbiota, immune system, innate immunity, therapy, and diet.

**Figure 9:**
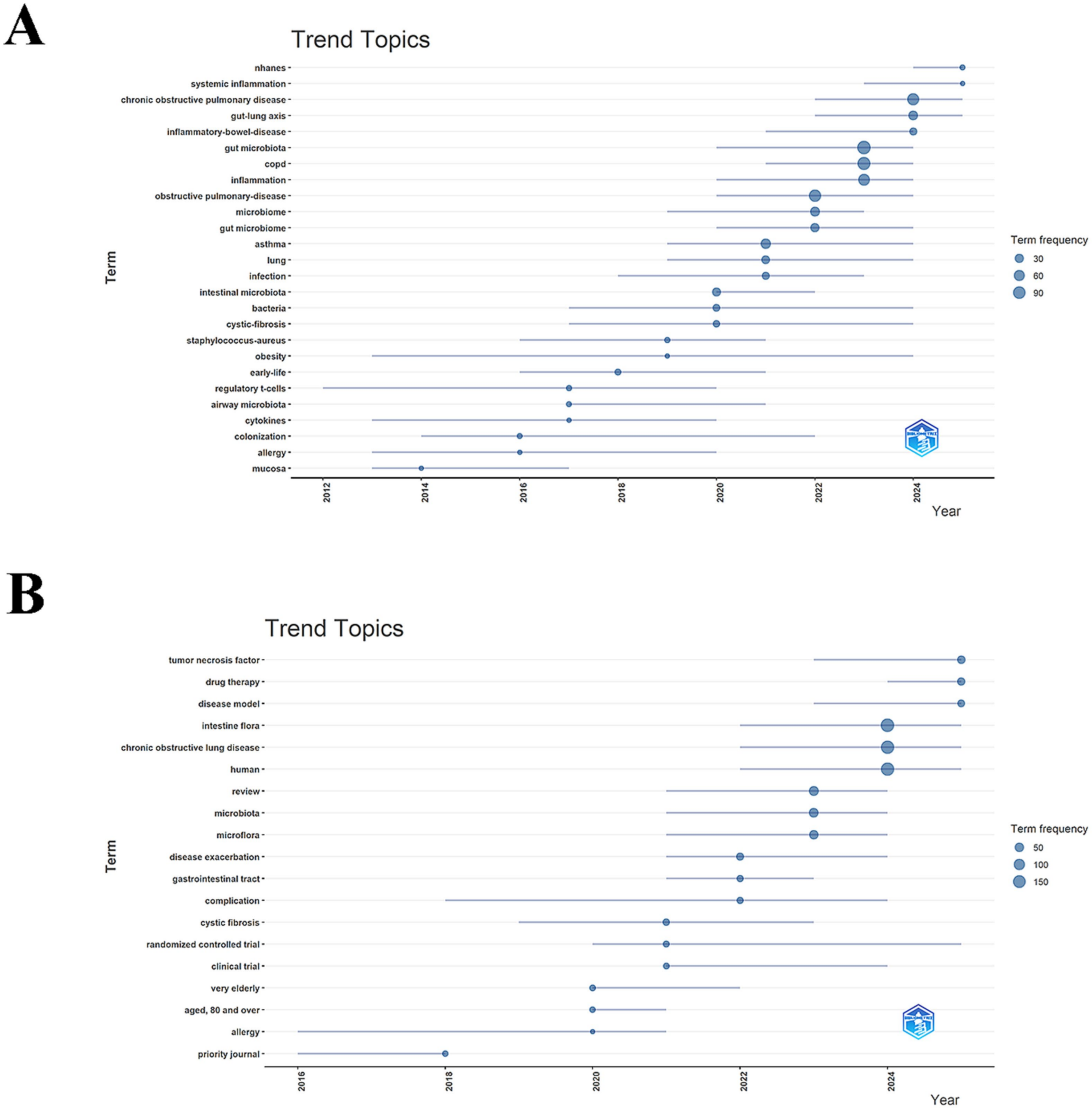
Trend topics on gut microbiota and COPD. **(A)** Trend topics based on WoSCC. **(B)** Trend topics based on Scopus.

To explore the evolutionary trends of research themes in this field, we used the bibliometrix package in R to construct thematic trend maps (Figure 9). Trend thematic maps facilitate tracking the temporal development of specific research topics, enabling monitoring of shifting research hotspots and deeper understanding of field progression. Analysis showed that from 2014 to 2017, research hotspots centered on fundamental topics such as “colonization,” “airway microbiota,” and “human gut microbiome,” indicating a primary focus on microbial community composition and early exposure. From 2018 to 2020, research gradually transitioned toward disease-related directions such as “gut microbiota” and “lung microbiome,” reflecting growing attention to links between microbiota and respiratory diseases. After 2021, research focus further narrowed to mechanistic and systemic themes including “chronic obstructive pulmonary disease,” “gut-lung axis,” and “short-chain fatty acids,” demonstrating deepening understanding of microbiota roles in host immune regulation and systemic diseases. Notably, the frequent emergence of “gut-lung axis” and “short-chain fatty acids” in recent years indicates a shift from descriptive studies to functional mechanism investigations closely tied to clinical disease management. Overall, the interaction between gut and lung microbiota and their roles in chronic inflammatory diseases have become core trends in current research.

Results from the Scopus database (Figure 9B) showed that research themes evolved from basic exploration of respiratory mucosal biology and microbial colonization toward mechanistic studies of gut microbiota-lung disease associations, ultimately focusing on pathological mechanisms and clinical translation of gut-lung axis regulation in COPD. Early studies from 2012 to 2016 focused on fundamental associations between respiratory mucosal characteristics, microbial colonization patterns, and allergic diseases, with keywords including mucosa, colonization, and allergy. Mid-term studies from 2017 to 2021 investigated cross-organ links between gut microbiota and respiratory diseases, establishing the conceptual framework of the gut-lung axis, with terms such as intestinal microbiota, lung, bacteria, and airway microbiota. From 2022 to the present, research has centered on gut microbiota, COPD, chronic obstructive pulmonary disease, inflammation, and gut-lung axis, emphasizing regulatory roles and targeted interventions of the gut microbiota-gut-lung axis in COPD pathogenesis and progression. Core terms such as gut-lung axis and inflammation further confirm a shift toward precise mechanistic dissection, multi-organ crosstalk, and clinical translation of gut microbiota regulation in COPD.

In summary, keyword and thematic trend analyses demonstrate that research on gut microbiota and its metabolites regulating COPD via the gut-lung axis has evolved from early basic exploration of respiratory mucosal biology, microbial colonization, and allergic disease associations to cross-organ linkage analysis between gut microbiota and respiratory diseases, establishment of the gut-lung axis conceptual framework, and characterization of disease-specific microbiome profiles. Current research is advancing deeply toward precise immune-inflammatory regulatory mechanisms of the gut microbiota-gut-lung axis, identification of microbiota intervention targets, screening of early warning biomarkers for COPD exacerbations, and clinical translational applications.

### 3.6 Integrated analysis of research hotspots

Combining citation bursts, keyword clustering, and thematic evolution analyses, this study identified four major research hotspots: 1. Lung and gut microbiota characteristics in COPD patients, including compositional changes in lung and gut microbiota across different COPD stages, severity levels, and inflammatory phenotypes; 2. Reciprocal interactions and mechanisms between COPD and gut microbiota, focusing on core signaling pathways of the bidirectional vicious cycle of the gut-lung axis (bacterial translocation, mucosal immune imbalance, pro-inflammatory cytokine cascades, etc.); 3. Gut microbiota-based intervention strategies for COPD, including exploration of high-fiber dietary modulation, specific probiotic supplementation, multi-target traditional Chinese medicine interventions, fecal microbiota transplantation, and other applications; 4. Immune mechanisms of gut microbiota metabolites mediating gut-lung axis regulation in COPD, focusing on short-chain fatty acids (SCFAs), trimethylamine N-oxide (TMAO), tryptophan metabolites, and secondary bile acids as key mediators.

## 4 Discussion

### 4.1 Basic information

This study established a comprehensive dataset consisting of 287 publications from the WoSCC database and 202 publications from the Scopus database published between 2009 and 2025. The results showed that the annual number of publications in this field exhibited an overall sustained growth trend, especially entering a stage of rapid rise after 2019, indicating that research on gut microbiota and COPD has become a research hotspot in the fields of respirology and microbiomics. An in-depth analysis identified two main dimensions of core driving factors for the rapid development of this field: first, governments and authoritative scientific research funding institutions in many countries around the world have continuously increased research investment in this field, and successively launched special research programs and major funded projects related to the human microbiome and the prevention and control of chronic respiratory diseases. For example, the Integrative Human Microbiome Project initiated by the National Institutes of Health (NIH) of the United States (Integrative HMP (iHMP) Research Network Consortium, 2019), the National Key R&D Program of China entitled Study on the Impacts and Mechanisms of Bacterial and Viral Infections on Acute Exacerbation of Chronic Obstructive Pulmonary Disease (Hua et al., 2020), and the Microbiome Program of the Chinese Academy of Sciences named Research on Common Technologies of the Microbiome for Human and Environmental Health etc. These research projects have funded a number of basic and clinical studies focusing on the regulatory mechanisms of the gut-lung axis, the role of gut microbiota in the occurrence and development of COPD, and microecological intervention targets, providing critical financial support and policy guidance for the development of this field. Second, in recent years, the series of reports by the Global Initiative for Chronic Obstructive Lung Disease (GOLD) have gradually clarified the potential role of the host microbiome in the progression and acute exacerbation of COPD, and the development of metagenomic technologies has further promoted the large-scale and systematic implementation of relevant research worldwide (Sharma et al., 2024).

#### 4.1.1 Countries/regions and institutions

China ranks first in the world in the cumulative number of publications in this field, followed by the United States, with the publication output of both countries far exceeding that of other countries and regions. This reflects the extremely high attention and investment of researchers from the two nations in studies related to gut microbiota and COPD. Among the world’s top 10 research institutions by publication output, the University of Technology Sydney in Australia ranked first. Among the other listed institutions, the WoSCC database showed 3 from China, while the Scopus database indicated 7 from China, which fully confirms China’s academic leading position in this field. The formation of this research pattern is closely linked to the prevalence and severity of COPD in China: epidemiological data show that the prevalence of COPD among people aged 60 and above in China exceeds 27%, with the total number of patients nationwide approaching 100 million (Chen B. et al., 2026; Xu et al., 2026). Thus, there is an increasingly urgent demand for research on novel therapeutic and management strategies for COPD prevention and control. From the perspective of the core characteristics of the academic cooperation network, iconic in-depth collaborative relationships have been formed among the top institutions in the field, with the long-term and close cooperation between the University of Technology Sydney and the University of Newcastle being the most representative. The research team led by Professor Philip M. Hansbro from the University of Technology Sydney and the team led by Professor Ian A. Yang from the University of Newcastle have conducted joint research. Through multi-omics sequencing analysis of fecal samples from COPD patients and healthy individuals as well as independent cohort validation, they identified the characteristics of gut microbiota and metabolome associated with COPD, screened out differential bacterial species linked to the decline in lung function, and constructed a disease-associated microbiota-metabolite regulatory network. This work has provided critical evidence for mechanistic research on the gut-lung axis in COPD and the discovery of novel biomarkers (Bowerman et al., 2020). Notably, Professor Lisa G. Wood has served as President of the Nutrition Society of Australia, and Professor Philip M. Hansbro is one of the core founders of the global gut-lung axis research consortium and an authoritative scholar in this field. Their academic influence and resource accumulation have laid a solid foundation for the in-depth cooperation between the two institutions. In addition, although Chinese academic institutions lead in publication volume, they have not formed an extensive international cooperation network. This also indicates that there remains considerable room for expansion and deepening in research on gut microbiota and COPD.

#### 4.1.2 Journal analysis

Regarding journal distribution, the 287 publications from the WoSCC database were distributed across 185 academic journals, and the 202 publications from the Scopus database were distributed across 137 academic journals. The core publishing journals mainly included the *International Journal of Chronic Obstructive Pulmonary Disease* and *Frontiers in Microbiology*. All the above journals are ranked in JCR Q1–Q2, suggesting that research in this field is generally of high academic quality. Further analysis of publication output and citation profiles of the journals showed that the *International Journal of Chronic Obstructive Pulmonary Disease* ranked first in the cumulative number of included publications in this study, while *PLOS ONE* ranked first in total citation frequency, which fully establishes their core academic position in the field of gut microbiota and COPD research. Notably, several top high-impact journals in this field exhibit a characteristic of “low publication proportion but high citation frequency.” The main reason for this feature is that such top journals have extremely rigorous peer-review processes and strict acceptance criteria. The studies they publish mostly focus on cutting-edge scientific issues and interdisciplinary innovative explorations, with strong theoretical innovation and clinical guiding value. Therefore, even though they are not the main source journals of the literature included in this study, their published achievements still serve as key theoretical support for advancing the development of the field and have been widely concerned and highly cited by researchers worldwide.

#### 4.1.3 Authors

An extensive and close academic cooperation network has been formed among researchers in this field. Among them, scholars including Hansbro, Philip M., Lee, Sei Won, Budden, Kurtis F., Gellatly, Shaan L., and Hugenholtz, Philip exert profound academic influence in the field, with the most outstanding research contribution from Hansbro, Philip M. A landmark representative achievement of the team led by Hansbro, Philip M. is an influential review published in Clinical & Translational Immunology in 2017. This review clarified the core roles of the respiratory and gut microbiomes in chronic respiratory diseases, dissected the immune regulatory mechanisms of the gut-lung axis, and revealed the key immune functions of microbial metabolites (Shukla et al., 2017). In 2021, the team demonstrated the anti-inflammatory, antioxidant, and immune-regulatory effects of various nutrients in multiple lung diseases, dissected their molecular mechanisms targeting key pathological signaling pathways, and proposed innovative nano-delivery strategies to improve the bioavailability of nutrients (Alemao et al., 2021; Gosens et al., 2020). In 2022, the team focused on SCFAs, the core metabolites of gut microbiota, and systematically elucidated their molecular mechanisms in regulating pulmonary immune homeostasis, protective effects against lung diseases, and clinical translation pathways (Ashique et al., 2022; Budden et al., 2022). In summary, the series of studies by the Hansbro, Philip M. team span the construction of the gut-lung axis theory, dissection of immune mechanisms, establishment of nutritional intervention systems, and design of clinical translation. They have built a multi-dimensional evidence chain for the prevention and treatment of lung diseases through microbiome-metabolite-nutritional intervention, providing core theoretical guidance for the clinical translation of this field.

Highly cited papers reflect the most valuable and influential research achievements and can further guide research directions in specific fields. The paper titled *The immune response to Prevotella bacteria in chronic inflammatory disease*, published in *Immunology*, is the most frequently cited article, with a total citation frequency of 1,071. This study confirmed that increased abundance of prevotella at mucosal sites mainly drives Th17-mediated mucosal and systemic inflammation through the activation of Toll-like receptor 2, contributing to various chronic inflammatory diseases (Larsen, 2017). In the Scopus database, the article *The Continuum of Aging and Age-Related Diseases: Common Mechanisms but Different Rates* published in *Frontiers in Medicine* was the most highly cited, with a total of 742 citations. This article discussed that aging, age-related chronic diseases, and geriatric syndromes share core biological mechanisms and all belong to a continuum without clear boundaries. Age-related diseases can be defined as accelerated aging, whose developmental trajectories are jointly determined by genetics, environment, and lifestyle. DNA methylation and other indicators can serve as key markers for the assessment of biological age (Franceschi et al., 2018).

Analysis of references with the strongest citation bursts can identify the most influential publications in the field. The main citation burst period in this field occurred from 2012 to 2024, covering the characteristics of changes in lung and gut microbiota in COPD patients, microecological interventions, probiotic regulatory mechanisms, and clinical effects. The paper *Emerging pathogenic links between microbiota and the gut-lung axis*, published in *Nature Reviews Microbiology* in 2017, had the highest citation burst strength. This review on the pathogenic links between microbiota and the gut-lung axis found that dysregulation of gut and respiratory tract microbiota can regulate immune responses through the gut-lung axis and participate in the occurrence and development of various lung diseases such as asthma, chronic obstructive pulmonary disease, and respiratory infections, suggesting that targeted regulation of gut microbiota may provide new intervention strategies for the prevention and treatment of lung diseases (Budden et al., 2017). The second study with the highest citation burst strength, a microbiome investigation of lung tissue from patients with very severe COPD, found that detectable low-abundance bacterial communities exist in human lung tissue. Although the total abundance and diversity of microbiota did not change significantly in patients with very severe COPD, the community structure showed characteristic changes and the abundance of Lactobacillus was significantly increased, indicating that alterations in lung tissue microbiota composition are closely related to the disease progression of very severe COPD (Sze et al., 2012). This translational review on the bidirectional role of the gut-lung axis in COPD found that COPD and cigarette smoking can induce intestinal inflammation, barrier damage, microbiota dysbiosis, and metabolic abnormalities, while intestinal lesions can further aggravate lung lesions through the bidirectional interaction of the gut-lung axis, providing a core theoretical basis for the development of novel adjuvant therapeutic regimens for COPD (Wang et al., 2023). In addition, papers still in the citation burst stage indicate that the field has thoroughly elucidated the key roles of gut microbiota dysbiosis and abnormal short-chain fatty acid metabolism in the pathogenesis of COPD, clarified the mechanism by which microbiota dysbiosis aggravates lung pathological damage through the gut-lung axis, identified the protective effects of beneficial microbiota and active components, and established targeted intestinal intervention directions, providing core theoretical and practical bases for the prevention and treatment of COPD (Cao et al., 2024; Lai et al., 2022; Otake et al., 2025).

### 4.2 Research hotspots and trends

Analysis of keywords and thematic trends can effectively reveal the core topics, hot directions, and evolutionary dynamics of a specific research field, providing an important basis for researchers to construct a domain knowledge framework and explore potential research directions. Based on the analysis of high-frequency keywords, keyword co-occurrence, keyword clustering, and thematic trends in the field of gut microbiota and COPD, the core research content of this field at the present stage can be systematically clarified.

#### 4.2.1 Characteristics of lung and gut microbiota in COPD patients

Through keyword co-occurrence analysis and visualization maps, a large number of microbial names such as “gut microbiota,” “lung microbiota,” “*Porphyromonas gingivalis*,” “*Lactobacillus casei*,” and “Lactobacillus,” can be identified, suggesting that various bacterial communities have always been the focus of attention in this field. Studies have shown that patients with COPD exhibit lung microbiota dysbiosis, mainly characterized by reduced diversity and increased abundance of pathogenic bacteria, which is associated with disease severity and inflammatory phenotypes 38,228,603. First, changes in the lung microbiota of patients with stable COPD mainly include increased abundance of Proteobacteria and decreased abundance of Bacteroidetes and Firmicutes (Yan et al., 2022). There are significant differences in lung microbiota composition among COPD patients of different severity levels: the Bacteroidetes level in the COPD grade III–IV group is lower than that in the healthy group or COPD grade I–II group, while the Firmicutes level is higher than that in the COPD grade I–II group (Li et al., 2021). The Prevotellaceae level is higher in the COPD grade I–II group, whereas the Bacteroidaceae and Clostridiaceae levels in the COPD grade III–IV group are lower than those in healthy individuals (Budden et al., 2019). Second, the inflammatory phenotypes of COPD are associated with distinct lung microbiota. The neutrophilic inflammatory phenotype is related to Proteobacteria, especially Haemophilus and Moraxella (Lan et al., 2023), while the eosinophilic inflammatory phenotype is associated with Firmicutes and Bacteroidetes, among which the Firmicutes-dominated microbiota is positively correlated with peripheral blood eosinophil count (Mayhew et al., 2018). Third, the composition of the lung microbiota also changes during acute exacerbation of COPD. Short-term antibiotic therapy reduces the proportion of Proteobacteria in the lung microbiota and restores the originally reduced diversity, which is beneficial for the treatment of acute exacerbations (Ni et al., 2026).

The composition of the gut microbiota in COPD patients also differs significantly from that in healthy individuals. The main changes in the gut microbiota of COPD patients are the reduction of Bacteroidetes and Roseburia (Pan et al., 2025; Chiu et al., 2021). Prevotella, Streptococcus Rothia, Romboutsia, and Enterobacteriaceae have been detected in the feces of COPD patients but not in healthy individuals (Bowerman et al., 2020). Studies have shown that changes in the gut microbiota of COPD patients may be associated with declined lung function. The reduction of Bacteroidetes is related to the decrease in forced expiratory volume in 1 s (FEV1) and forced vital capacity (FVC). The abundance of Prevotella and Streptococcus is negatively correlated with FEV1, and the abundance of Clostridium in COPD grade III–IV patients is higher than that in COPD grade I–II patients (Chiu et al., 2021). In addition to changes in gut microbiota composition, COPD patients also exhibit alterations in gut microbiota metabolites, mainly reduced levels of short-chain fatty acids (SCFAs) and elevated levels of lipopolysaccharide (LPS) (Kotlyarov, 2022). The decrease in SCFAs is associated with reduced Bacteroidetes abundance and increased Firmicutes abundance. SCFAs are saturated fatty acids produced by gut microbiota during the fermentation of dietary fiber, including butyrate, propionate, and acetate. As metabolites of gut microbiota, SCFAs benefit energy metabolism, protect intestinal barrier function, regulate immunity, and also participate in modulating airway epithelial function (Chen T. et al., 2026; Imoto et al., 2018). In summary, the lung and gut microbiota of COPD patients show a pattern of reduced diversity and increased pathogenic bacteria. Such microbiota imbalance not only affects COPD severity and inflammatory phenotypes but also correlates with declined lung function. Future research requires large-sample longitudinal cohort studies and standardized multi-omics analysis of microbiota to clarify the causal role of dynamic changes in lung and gut microbiota during COPD progression, as well as individual heterogeneity across different disease severity, inflammatory phenotypes, and stages. Meanwhile, the specific mechanisms of key microbiota and their core metabolites in regulating COPD inflammation and the progression of lung function injury should be further explored, so as to provide a more comprehensive theoretical basis for the precise prevention and treatment of COPD and the development of targeted microbiota intervention strategies.

#### 4.2.2 Interactive effects and mechanisms between COPD and gut microbiota

Through high-frequency keyword analysis, keyword clustering, and citation burst detection, we found that terms related to “gut-lung axis,” “cross-talk,” “interplay,” and “smoke exposure” represent important research topics in the field of gut microbiota and COPD. Many studies have confirmed the mutual influence between the gut and lungs. Mice receiving fecal transplantation from COPD patients developed lung inflammation, while COPD model mice transplanted with fecal microbiota from healthy individuals showed alleviated lung and intestinal inflammation (Li et al., 2021). Smoking is an important risk factor for COPD development (Otake et al., 2025). Studies have found that smokers have increased fecal abundance of Bacteroidetes, Prevotella, Escherichia, Shigella, and Klebsiella, and decreased abundance of Firmicutes and Proteobacteria (Antinozzi et al., 2022; Bai et al., 2022). These bacteria are important commensal bacteria in the gut that regulate intestinal barrier function and inflammatory and immune responses associated with intestinal diseases (Wastyk et al., 2021). Furthermore, COPD may impair intestinal barrier function, causing structural changes in the intestinal mucosa, hypoxia, inflammatory cell infiltration, local shedding of epithelial cells, and subsequent intestinal barrier damage and dysfunction (Xin et al., 2016). Gut microbiota and its metabolites participate in regulating intestinal barrier permeability and immune responses. Dysbiosis of gut microbiota leads to increased proportions of pathogenic bacteria and pro-inflammatory factors, oxidative stress, disruption of local immune homeostasis, and accelerated intestinal barrier dysfunction (Kou et al., 2025), (Fernandez-Cantos et al., 2021). Studies have found that intestinal barrier-related biomarkers such as citrulline, diamine oxidase, and D-lactate dehydrogenase can be used to assess COPD severity (Yin et al., 2021). In addition, the proportion of colonized Gram-negative bacteria such as Klebsiella in the lungs of COPD patients is elevated, and LPS produced by Gram-negative bacteria can induce systemic immune and inflammatory responses, further affecting the gut microbiota (Yang et al., 2021).

Gut microbiota dysbiosis exerts adverse effects on intestinal mucosal barrier function and systemic immune responses. Impaired intestinal barrier function may lead to the translocation of bacteria and their products, among which bacterial toxins such as enterotoxin and LPS can elevate local and systemic inflammatory mediator levels, representing a key mechanism promoting the occurrence and progression of COPD (Cardenas et al., 2022; Tao et al., 2024). Gut microbiota dysbiosis may promote COPD through the following mechanisms: 1. Destruction of the intestinal mucosal barrier structure allows intestinal bacteria to migrate to the lungs via increased intestinal permeability and mesenteric lymphatic vessels (Dickson et al., 2016). 2. Gut microbiota dysbiosis causes decreased immunity. Mucosal tissues of both the gastrointestinal and respiratory tracts secrete large amounts of secretory immunoglobulin A (sIgA), which neutralizes pathogens to protect the host (Zhang et al., 2025). sIgA deficiency is common on the airway surface of COPD patients (de Fays et al., 2022), and the synthesis and secretion of sIgA in both the lungs and intestines are reduced in COPD patients with infection (Han et al., 2011). 3. Gut microbiota dysbiosis promotes the secretion of pro-inflammatory factors (such as IL-1β, IL-8, TNF-*α*, IFN-*γ*, etc.). These pro-inflammatory factors enter the lungs, activate and recruit inflammatory cells around the airways, damage alveolar cells, and disrupt the physiological structure of the lungs (Liu et al., 2025; Raftery et al., 2020). 4. Gut microbiota dysbiosis can affect the regulation of T helper cell 17 (Th17), regulatory T cells (Treg), the Th17 regulator retinoid-related orphan receptor-γt (ROR-γt), and the Treg marker forkhead box protein P3 (Foxp3), leading to immune abnormalities (Jia et al., 2024; Wang et al., 2024). 5. SCFAs can directly or indirectly protect lung function by regulating pulmonary immune homeostasis and intestinal barrier function. Gut microbiota dysbiosis reduces SCFA levels, weakens anti-inflammatory and immunoregulatory capacities, and further affects systemic and pulmonary inflammatory responses (Ashique et al., 2022; Trompette et al., 2014).

In summary, existing studies confirm the bidirectional regulatory role of the gut-lung axis in COPD: COPD can induce gut microbiota dysbiosis, and the dysregulated gut microbiota modulated by COPD further promotes COPD progression, forming a vicious cycle. However, most current studies only focus on changes in microbiota and cannot clarify whether gut microbiota dysbiosis is an initiating factor of COPD, a concomitant outcome of the disease, or a key amplifying link accelerating disease progression. The core causality and temporal sequence of the bidirectional regulation of the gut-lung axis remain to be clarified.

#### 4.2.3 COPD intervention strategies based on gut microbiota

Keyword analysis and visualization show that diet, dietary fiber, probiotics, and traditional Chinese medicine (TCM) are important research directions in this field suggesting that these interventions all target gut microbiota as a core regulator of COPD. They can target the gut-lung axis in multiple dimensions: On the one hand, increasing the abundance and composition of probiotics; on the other hand, inhibiting the proliferation of pathogenic bacteria. In addition, these interventions help maintain intestinal barrier function, ultimately improving COPD pathology, alleviating inflammatory responses, and achieving multi-dimensional benefits in microbiota-immunity-lung function (Ni et al., 2026)

Dietary regulation-based prevention and treatment strategies represent a potential non-pharmacological approach with no side effects for the prevention and treatment of COPD, respiratory diseases, and smoking-related cancers (Beijers et al., 2023). A higher Alternate Healthy Eating Index is associated with lower COPD risk. A prospective study of Swiss women found that long-term high dietary fiber intake was associated with a 30% reduction in COPD risk (Szmidt et al., 2020). High intake of cereal and fruit fiber is associated with lower COPD risk, but not with vegetable fiber intake (Budden et al., 2024). A high-fiber diet mainly acts by reducing the Bacteroidetes/Firmicutes ratio in the gut microbiota and enhancing fiber metabolism, increasing circulating SCFAs. These metabolites systemically inhibit HDACs, activate GPCRs (GPR41, GPR43), and suppress NF-κB-driven inflammatory pathways in the lungs, thereby enhancing intestinal barrier function, regulating systemic immunity, and improving lung function (Jang et al., 2021).

Probiotics can actively regulate host immunity and pathogenic microbiota, showing benefits in improving lung function (Yu et al., 2025). Studies have found that *Lactobacillus rhamnosus* can restore the balance of pro-inflammatory and anti-inflammatory factors in human bronchial epithelial cells and alleviate inflammatory responses in the airways and lung parenchyma of smoke-induced COPD mice. Its mechanism is related to enhancing Treg responses and promoting SCFA production (Yan et al., 2022). In COPD models, intranasal administration of specific such strains restores indole-3-acetic acid (IAA) levels, activates the interleukin-22 (IL-22) signaling pathway, and simultaneously reduces neutrophilic inflammation and emphysematous lesions (Wang C. et al., 2025). Supplementation with acetate-producing *Bifidobacterium longum* subspecies alleviates pulmonary inflammation and the expression of inflammatory cytokines and adhesion factors (Budden et al., 2022). A prospective, multi-center, randomized trial showed that long-term oral administration of *Lactobacillus rhamnosus* GG significantly slowed the progression of COPD from mild to severe (Hua et al., 2024; Vasconcelos et al., 2025).

TCM and new compound preparations provide multi-target intervention strategies for regulating the gut-lung axis (Zheng et al., 2025). “Qipian” has shown good efficacy in COPD models: it increases the abundance of beneficial microbiota (Bacteroidetes, Lactobacillus) and inhibits the TLR4/NF-κB signaling pathway, thereby reducing pro-inflammatory cytokine levels, alleviating alveolar structural damage, and improving lung function (Sun et al., 2025). The TCM compound Qifeng Gubiao Granules (QFGB) can regulate gut microbiota, restore the polarization of intestinal M1 and M2 macrophages to alleviate intestinal inflammation, and improve lung tissue injury by alleviating oxidative stress and ferroptosis in lung tissue via the gut-lung axis, exerting a therapeutic effect on COPD mice (Kang et al., 2026). Clinical trials have shown that TCM interventions (such as Compound Caoshican Granules, Xuanbai Chengqi Decoction, etc.) can improve symptoms in COPD patients and reduce the frequency of acute exacerbations, and their efficacy is closely related to gut microbiota regulation (Liu K. et al., 2026; Yu et al., 2026).

In summary, non-pharmacological interventions and natural product therapies targeting gut microbiota regulation have gradually emerged as pivotal research priorities in this field. Diet, probiotics, and TCM represent the three core intervention strategies and collectively constitute the main research backbone of this research area. All three approaches take gut microbiota as the central regulatory target, and a multidimensional theoretical framework of “microbiota–immunity–pulmonary function” has been well established. Future research should precisely screen probiotic strains and active TCM components with specific therapeutic effects on COPD, and improve the applicability of intervention regimens for distinct populations, such as obese and elderly individuals. Such efforts will substantially advance the clinical translation and practical application of relevant findings.

#### 4.2.4 Gut microbiota metabolites and COPD: key mediators of immune regulation

In recent years, the immune regulation of lung function mediated by gut microbiota through metabolites, including SCFAs, trimethylamine N-oxide (TMAO), tryptophan metabolites, and secondary bile acids, has gradually become a research hotspot.

SCFAs are mainly produced by commensal gut microbiota through the fermentation of indigestible dietary components ingested by the host, and they also play a key role in maintaining pulmonary physiological homeostasis. Studies have found that COPD severity is associated with reduced fecal SCFA concentrations. SCFAs can regulate Treg generation and polarize lung macrophages toward an anti-inflammatory M2 phenotype by inhibiting histone deacetylase (HDAC) and signal transduction, thereby improving pathological morphological damage to lung tissue (Jiang et al., 2024; Li et al., 2025). Meanwhile, butyrate may also significantly alleviate pulmonary inflammatory infiltration and mucus hypersecretion phenotype in a mouse model of ovalbumin (OVA)-induced type 2 airway hyperresponsiveness by targeting and suppressing Th9 cell-mediated immune responses (Vieira et al., 2019). In addition, during acute exacerbation of COPD, butyrate treatment reduces the proportion of inflammatory ILC_2_ cells and the secretion of inflammatory factors in colon and lung tissue (Jiang et al., 2023). As an important energy source for colonic epithelial cells, SCFAs can enhance intestinal barrier function, thereby effectively inhibiting the spread of pathogens and LPS in the intestinal lumen (Zheng et al., 2025). In summary, existing studies have fully confirmed that SCFAs are closely related to the occurrence, pathological progression, and acute exacerbation of COPD.

TMAO, a key gut microbiota-derived metabolite, has been shown to have a significant positive correlation between elevated circulating TMAO levels and all-cause mortality risk in COPD patients (Ottiger et al., 2018); moreover, TMAO may serve as a potential biomarker for evaluating poor prognosis in COPD patients (Suzuki et al., 2019). TMAO is mainly involved in COPD progression by inducing pulmonary vascular structural injury through mediating inflammatory responses. Existing studies suggest that TMAO may indirectly mediate pulmonary vascular remodeling by activating macrophages and driving the release of pro-inflammatory mediators and chemokines (Liu J. et al., 2026; Yang et al., 2024). Current understanding of the role of TMAO in the pathogenesis of COPD and other chronic lung diseases remains limited, and the relevant molecular regulatory pathways and clinical translation value have not been fully elucidated.

Tryptophan metabolites (including indole-3-acetic acid, IAA) participate in the regulation of pulmonary immunity. IAA produced by Lactobacillus inhibits the production of pro-inflammatory cytokines; kynurenine can activate the aryl hydrocarbon receptor (AhR) on the surface of T cells, promote Treg differentiation and IL-22 secretion, and further enhance epithelial barrier integrity (Fong et al., 2023). During COPD progression, depletion of tryptophan-metabolizing gut microbiota reduces IAA and kynurenine levels, thereby impairing AhR signaling (Solvay et al., 2023). Bile acids modified by gut microbiota also participate in the signal regulation of the gut-lung axis. Tauroursodeoxycholic acid (TUDCA), a secondary bile acid, can inhibit the activation of the NLRP3 inflammasome in macrophages but may also impair neutrophil function, increasing the risk of infection (Wang L. et al., 2025).

In conclusion, the clinical translational value of gut microbiota metabolite research has become increasingly prominent. SCFAs signaling pathways, TMAO, and secondary bile acids collectively form an abundant pool of regulatory targets with great potential for drug development within the COPD gut–lung axis. Nevertheless, current studies predominantly focus on SCFAs, while research on tryptophan metabolites, secondary bile acids, and other functional metabolites remains insufficient. In addition, existing mechanistic investigations tend to be overly simplistic and linear. Future research should adopt human-relevant models such as organoid chips to validate the above mechanisms and accelerate the development of novel targeted therapeutics, including metabolite-based biological agents and intestine/lung-targeted nanodelivery systems. In-depth and systematic clarification of the regulatory mechanisms underlying the gut–lung axis is critical for the development of innovative therapeutic strategies for COPD.

### 4.3 Differences between the WoSCC and Scopus databases and their impacts on research hotspot identification

This study performed bibliometric analysis using both the WoSCC and Scopus databases to systematically delineate the overall knowledge structure and developmental trajectory of research on gut microbiota and COPD from complementary perspectives. Notably, this study did not assume equivalence between the two databases in terms of publication volume, disciplinary coverage, and research types. Instead, we fully considered their long-standing structural differences and deliberately adopted a dual-database strategy to improve the comprehensiveness and robustness of the findings. In this analysis, the number of relevant publications retrieved from the WoSCC database was higher than that from Scopus (287 vs. 202 articles). This discrepancy mainly stemmed from systematic differences in journal selection criteria and disciplinary coverage between the two databases: WoSCC places greater emphasis on journal academic impact and stability, prioritizing literature from high-impact journals with solid theoretical foundations and rigorous methodologies. In contrast, Scopus provides broader coverage of clinical medicine, public health, and applied research, and is more inclusive of publication types such as clinical studies, observational studies, and case reports. As an emerging research hotspot in recent years, investigations into gut microbiota and COPD are currently strongly basic-science-oriented, whereas clinical research remains in the initial stage. Moreover, basic research is often conducted using animal experiments, so it was expected that WoSCC would capture more relevant publications. Such database-specific characteristics were also reflected in the keyword analysis: compared with Scopus, the WoSCC dataset contained more high-frequency keywords related to basic research, while the Scopus dataset was biased toward keywords associated with clinical practice, case observation, and study designs, with more refined clinical terminology including human, aged, female, adult, and clinical outcome (Table 9; Figure 8). This pattern does not indicate a shift in research themes, but highlights the advantage of Scopus in capturing real-world clinical research and early exploratory studies.

### 4.4 Limitations

This study aims to deepen the understanding of the research status and hotspots of gut microbiota in the field of COPD, and to explore valuable potential research directions. However, this study still has several limitations. First, this study only included data from the WoSCC and Scopus databases, which may lead to the omission of literature published exclusively in other databases. Second, only English-language literature was analyzed in this study, which may neglect research conducted in other languages and introduce data source bias. Third, in terms of document types, only reviews and original articles were included, which may also result in data omission. Despite these limitations, this study provides a comprehensive overview of the field and clarifies its core themes and developmental trends. By considering the above factors, researchers can gain a deeper insight into the overall progress of the field, identify potential research directions, and provide references and guidance for future investigations.

## 5 Conclusion

This study reviewed the key research hotspots and emerging frontiers in the field of gut microbiota and COPD. The main intellectual context and research trends in this field are summarized as follows:

- Research related to gut microbiota and COPD has received extensive global attention. China and the United States are the core research forces in this field, with high research activity and close international cooperation. With the continuous deepening of relevant research, international collaboration will be further strengthened in the future.
- In this field, International Journal of Chronic Obstructive Pulmonary Disease has the largest number of publications, fully reflecting its representative status as a core academic platform in the field.
- Co-authorship analysis of literature on gut microbiota and COPD shows extensive and close cooperation among researchers, with Hansbro, Philip M. being a representative core author in this field.
- The characteristics of gut microbiota changes in COPD patients have become an important research hotspot in this field.
- The interactive effects and mechanisms between COPD and gut microbiota represent a key research hotspot and development trend in this field.
- Exploring microecological intervention strategies—including diet, probiotics, traditional Chinese medicine, and fecal microbiota transplantation—has become an important research trend in this field. In addition, immune regulation mediated by gut microbiota metabolites has emerged as a new hotspot.

In summary, this study provides a valuable reference for the analysis of research trends and hotspots in the field of gut microbiota and COPD. The above results help researchers quickly grasp the current research status of this field and explore future directions. This study also points out the limitations of current research and potential key directions, providing guidance for researchers to deepen relevant studies and develop innovative research ideas.

## Data Availability

The datasets presented in this study can be found in online repositories. The names of the repository/repositories and accession number(s) can be found in the article/Supplementary material.
